# Soloxolone methyl induces apoptosis and oxidative/ER stress in breast cancer cells and target cancer stem cell population

**DOI:** 10.55730/1300-0152.2660

**Published:** 2023-06-05

**Authors:** Elif ERTÜRK, Oğuzhan AKGÜN, Yaren YILDIZ, Pınar ALPER KALKAN, Oksana V. SALOMATINA, Nariman F. SALAKHUTDINOV, Engin ULUKAYA, Ferda ARI

**Affiliations:** 1 Vocational School of Health Services, Bursa Uludağ University, Bursa, Turkiye; 2 Department of Biology, Faculty of Science and Arts, Bursa Uludağ University, Bursa, Turkiye; 3 Aziz Sancar Experimental Medicine Research Institute, Molecular Medicine, İstanbul University, İstanbul, Turkiye; 4 N.N. Vorozhtsov Novosibirsk Institute of Organic Chemistry, Siberian Branch of the Russian Academy of Sciences, Novosibirsk, Russia; 5 Department of Clinical Biochemistry, Faculty of Medicine, İstinye University, İstanbul, Turkiye

**Keywords:** Mammosphere, soloxolone methyl, apoptosis, breast cancer

## Abstract

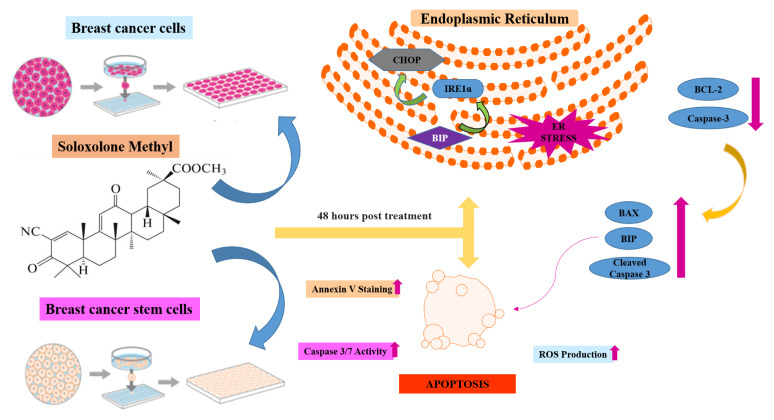

One of the most prevalent malignancies in women and one of the leading causes of cancer-related death is breast cancer. There is a need for new treatment approaches and drugs for breast cancer. Many studies show the high potential of triterpene compounds and their semisynthetic derivatives as anticancer agents due to their ability to induce apoptosis and suppress tumorigenesis. The effects of soloxolone methyl (SM), a semisynthetic derivative of 18-H-glycyrrhetinic acid, on the cytotoxicity and apoptosis of human breast cancer cell line (T-47D) and cancer stem cell (CSCs) population (mammospheres; CD44+/CD24-antigen) derived from breast cancer cells, were examined in this work. The ATP assay was used to determine SM growth-inhibitory effects. Fluorescent staining, caspase-cleaved cytokeratin 18, and flow cytometry analysis were used to determine the mode of the cell death. In addition, cell death was investigated at protein and gene levels by Western Blotting and PCR, respectively. SM resulted in cytotoxicity in a time and dose dependent manner via ROS production and ER stress in T-47D cells in 2 models. The mode of cell death was apoptosis, evidenced by phosphatidylserine exposure, caspase activation, and bax overexpression. In mammospheres as 3D model, SM decreased stem cell properties and induced cell death. Taken together, SM may be a promising agent in the treatment of breast cancer, especially due to its antigrowth activity on CSCs.

## 1. Introduction

One of the biggest causes of mortality and morbidity in the globe is cancer. It is accepted as one of the most common diseases in the world after cardiovascular diseases, and its incidence increases every year (Ames et al., 1995). According to the data of the International Cancer Research Center (IARC) and the World Health Organization (WHO), 18.1 million new cancer cases and 9.6 million cancer deaths were recorded in 2018 (Ferlay et al., 2019). The most frequently diagnosed cancer in 2018 was lung cancer in men (14.5%) and breast cancer (BC) in women (24.2%) (Ferlay, 2019). In the entire world, BC is the most prevalent type of cancer in women and the leading cause of cancer-related death in females. Although many developments in cancer diagnosis and treatment have been reflected in BC, survival rates are still low due to this disease (Feng et al., 2018).

BC patients are treated with surgery, chemotherapy, radiotherapy, and hormone treatments (called antiestrogen therapies). Unfortunately, resistance to these types of therapy develops in 30%–40% of patients, and metastases may occur in the future (Klinge, 2018; Piggott et al., 2018). Therefore, there is an urgent need to elucidate the mechanisms underlying the ineffectiveness of current treatments in BC, and to develop new treatment options and new drugs. Previous studies support the CSCs hypothesis, which argues that breast cancer develops by populations of cells displaying stem cell characteristics. According to this hypothesis, CSCs are defined as cells that have self-renewal, specific organ, or tissue-specific characteristics, and can transform into many different cell types. In addition, studies prove that CSCs are resistant to chemotherapy and radiotherapy and therefore increase the risk of recurrence and metastasis in BC (Liu et al., 2012).

Discovery of biomarkers that define CSCs and validation studies in mouse models with in vitro experiments allow isolation and characterization of stem cells from tumor cells. In a study of stem cells in BC, it was shown that cells were shown to express ESA and CD44 but were negative for CD24 expression (Liu et al., 2012). In another study, approximately 200 cells with ESA(+), CD44(+), and CD24(−) were able to form tumors in immune system suppressed NOD/SCID mice; however, a 100-fold greater number of cells isolated from the same tumor that did not express cell surface markers proved to be nontumorigenic (Al-Hajj et al., 2003). Other researchers have commented on ALDH (aldehyde dehydrogenase) gene expression for the identification and enrichment of CSCs populations that initiate tumorigenesis in BC (Ginestier et al., 2007). All of these findings back up theories and assessments about breast cancer stem cells (BCSCs) resistance to chemotherapy and radiation therapy (Phillips et al., 2006; Shafee et al., 2008; Korkaya et al., 2009). In addition, Li et al. state that the increase in the percentage of BCSCs following neoadjuvant chemotherapy indicates therapeutic resistance and that successful approach targeting BCSCs are needed in treatment (Li et al., 2007).

**Figure 1:**
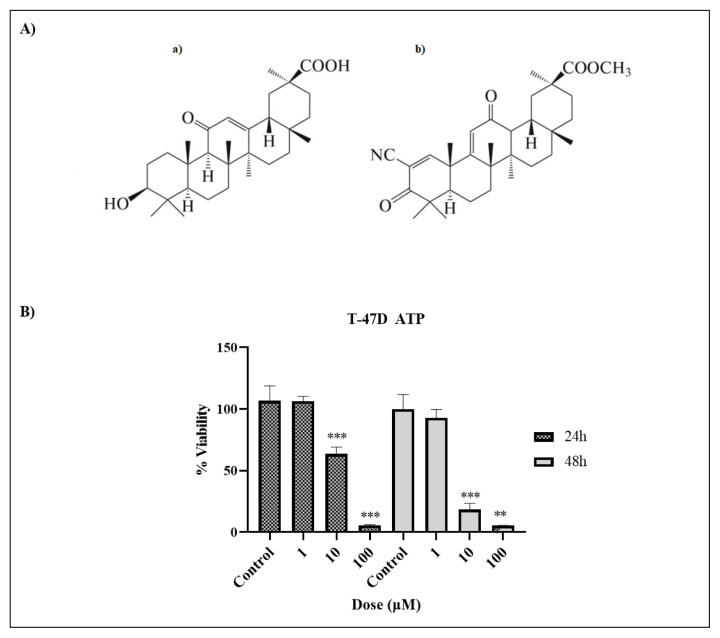
(A) Chemical structure of 18βH-Glycyrrhetinic acids (a) and SM (b) (Markov et al., 2019). (B) Viability of T-47D cell treated with SM for 24 and 48 h. ATP assays were performed as cell viability assays. *Indicates statistically significant differences compared to untreated control: ***(p < 0.001). Data are presented as mean ± SD (n = 3).

Plants have an important place in the discovery of anticancer drugs and their use in cancer treatment has a long history. Triterpenoids are a class of molecules with over 100 distinct skeletal configurations, all of which have a 30-carbon isoprene skeleton. The majority of plant secondary metabolites contain them (Connolly and Hill, 2002). *Glycyrrhiza glabra* (Commonly known as European licorice), *Glycyrrhiza uralensis* Fisch, and *Glycyrrhiza inflata* Bat all produce large amounts of the active triterpenoid metabolite 18H-Glycyrrhetinic acid (18H-GA) (used in the Chinese Pharmacopoeia) (Pastorino et al., 2018). Liquorice root was advised by Chinese medicine practitioners to help people live longer, heal injuries, and detoxify reactive oxygen species (Nomura and Fukai, 1998; Wang and Nixon, 2001). Additionally, it has received FDA (Food and Drug Administration) approval for the inclusion of a variety of items as dietary supplements. The anticancer action of 18βH-GA was assumed to be due to tumor suppression and apoptosis induction (Hibasami et al., 2005; Zhu et al., 2015; Cai et al., 2017). When it comes to the biological activities of their molecular targets, triterpenoids, including 18βH-GA, have a minor impact. Therefore, these chemicals are employed as building blocks in the development of more potent analogues (Roohbakhsh et al., 2016). Soloxolone methyl (methyl 2-cyano-3,12-dioxo-18βH-olean-9(11),1(2)-dien-30-oate; SM) is a new semisynthetic derivative of 18βH-GA (Figure 1A) (Logashenko et al., 2011).

In human epidermoid carcinoma cells (KB-3-1), SM decreases viability in a dose-dependent way and triggers G2/M arrest and caspase-dependent death, according to a prior study (Logashenko et al., 2011). ER stress is a key player in the molecular events caused by SM in tumor cells, according to improved transcriptome analysis (Markov et al., 2019). In addition, in animal models of Krebs-2 carcinoma, SM displays both antiinflammatory and antitumor properties (Markov et al., 2018). Our group’s area of interest is focused on investigating the effect of SM on breast cancer. Thus, SM was discovered to be toxic by inducing apoptosis in MCF-7 and MDA-MBA-231 breast cancer cells. It has been found that ER stress causes apoptosis, particularly in MDA-MB-231 cells (Alper et al., 2021). Here, we looked into the cytotoxic and apoptotic effects of SM on the T-47D breast cancer cell line and BCSCs, as well as its mode of action.

## 2. Materials and methods

### 2.1. Chemicals and cell culture

The T-47D cell line differs from other human breast cancer cells in that progesterone receptors are not regulated by estradiol, a hormone abundant in cells. T47D cells are extremely resistant to estrogens and antiestrogens. Estradiol does not stimulate progesterone receptors, and concentrations of naphoxide, which are cytotoxic to estrogen receptor-positive cells, have no effect on cell growth or progesterone receptor levels (Horwitz et al., 1982). T-47D breast cancer cells were grown as monolayers in DMEM/F-12 (1:1) medium supplemented with 10% heat-inactivated fetal bovine serum (Gibco, USA), 1% L-glutamine (Life Technologies, USA), and 0.5 percent primocin (Invivogen, USA) at 37 °C in a humidified environment containing 5% CO_2_. The SM chemical synthesis has been previously described (Logashenko et al., 2011). A stock concentration of the SM chemical (100 mM) was created in DMSO (the final concentration of DMSO is 0.1 percent). In full culture media, successive dilutions from 1 to 100 M were created.

### 2.2. Determination of antigrowth effect of SM in T-47D cell apoptosis assays

For ATP testing, 1, 10, and 100 μM concentrations of SM compound prepared in 96-well cell culture dishes were applied to T-47D cells for 24 and 48 h. As an indirect measure of the number of live cells, the luminogenic ATP assay calculates the amount of cellular ATP (Ulukaya et al., 2008). SM treatment was applied in T-47D cell line at concentrations of 100, 10, and 1 μM for 24 and 48 h. A luminometer (Bio-Tek, Multi-Mode Microplate Reader, USA) was used to measure the luminescence signal, and the result was expressed as relative light units (RLU). The viability of the treated cells was determined using the formula viability (percent) = 100 × (Sample Abs-Blank)/(Control Abs-Blank) with reference to untreated control cells, based on the absorbance findings of the ATP test. (Blank: It is the solution used to set the absorbance to zero before the sample is read in the spectrophotometer).

### 2.3. Fluorescent imaging for determination of cell death mode in T-47D cells

Under cell culture conditions, fluorescent dyes are used to detect nuclear morphology and membrane integrity. Propidium iodide (PI) can only pass through damaged membranes, labeling only primary necrotic and late apoptotic (secondary necrosis) cells. SM treatment was administered at a dose of 10 μM in T-47D cell lines. At the end of an incubation period of 24, 48, and 72 h, the T-47D cell line was evaluated under a fluorescent microscope by staining with a working solution of 1 μg/mL PI as applied in another study of ours (Alper et al., 2019).

### 2.4. M30-antigen (caspase-cleaved cytokeratin 18) method

Caspases, an enzyme group that is only activated in apoptotic cells, cleave CK18, an important cytoskeletal protein during apoptosis, generating CK18 (CK18-Asp396). The Asp396 fragment of CK18 (M30-antigen) is uniquely recognized by the M30 monoclonal antibody, indicating that CK18 is an apoptotic marker (Erturk et al., 2021). To determine the M30-antigen fragment by ELISA, T-47D cells were treated with 10 μM SM compound for 24 and 48 h. After 24 and 48 h of incubation, 10 μL of 10% Triton-X 100 was added to all wells. It was incubated for 15 min on a 600 RPM shaker at room temperature. Supernatant from all wells was collected and centrifuged at 2000 rpm for 30 s and analyzed against the kit contents of the M30 Cytodeath ELISA (M30-Cytodeath ELISA kit, Peviva, Bromma, Sweden). The color intensity obtained at the end of the experiment was read spectrophotometrically at 450 nm (Bio-Tek, Multi-Mode Microplate Reader, USA).

### 2.5. Flow cytometry (caspase 3/7 activity, annexin V, oxidative stress)

T-47D cells were given a 10 μM dosage of SM and incubated for 24 h. Flow cytometry was used to look at caspase 3/7 activity, annexin V staining, and reactive oxygen species (ROS) generation. Untreated cells were employed as a control group. At the conclusion of the treatment time, the cells were resuspended and treated with antibodies tailored for the cell surface. Fluorescent dyes were used to identify these antibodies. The tagged cells were laser exposed after being pumped under pressure via a capillary tube known as a “flow cell”. The instrument and kit compatible with Muse TM Cell Analyzer (Millipore, Germany) were utilized (Done et al., 2022).

### 2.6. Confirmation of ROS-induced cell deaths with inhibitors

To further analyze the cell death mode, 5 mM oxidative stress inhibitor NAC (N-Acetylcysteine) was administered to the cells as a pretreatment (1 h). After the incubation period, SM was applied to T-47D cells for 24 and 48 h at doses deemed appropriate in the ATP test.

### 2.7. SDS–PAGE and western blotting

Western blot analysis is a special protein-protein hybridization technique used to show the presence of the sought (target) protein in a protein mixture obtained from cells and to determine its molecular weight. Apoptosis ((Cleaved Caspase-3, 1:1000, #9661, Cell Signaling Technology), (Pro-Caspase-3, 1:1000, #14220, Cell Signaling Technology), (Cleaved-PARP, 1:1000, #5625, Cell Signaling Technology), (BCL-2, 1:1000, #4223 Cell Signaling Technology), (BAX, 1:1000 #41162, Cell Signaling Technology)), ER-stress marker (IRE1-α, 1:1000, #3294, Cell Signaling Technology), (BİP, 1:1000, #3177, Cell Signaling Technology), (CHOP 1:1000, #2895, Cell Signaling Technology)) in T47-D cells treated with SM compound, related signaling pathway components were investigated at the protein level. Cells exposed to 10 μM SM treatment for 24 h and unexposed control cells were first lysed to reveal the proteins in them. Total protein amount was determined by BCA (bicinchoninic acid assay) method. Equal amount of protein (20 μg) for each sample was loaded onto 10% SDS-polyacrylamide gels and subjected to electrophoresis. Proteins were transported to membranes made of nitrocellulose. Membranes were blocked with 5% skim milk powder dissolved in PBS (PBS-T) containing 0.1% Tween-20 (nonspecific binding blockage). After treatment with the primary antibody, the membranes were washed 3 times with PBS-T. It was incubated with HRP-bound secondary antibody for 1 h. It was washed 3 times with the same buffer and visualized by chemiluminescence method.

### 2.8. RT-PCR assay in T-47D cells

After 48 h of SM treatment, T-47D were collected for RNA isolation. The Purelink RNA Mini kit (ThermoFisher, USA) was used to isolate cellular RNA as in our previous study (Akgun et al., 2019). The Transcriptor First Strand cDNA Synthesis Kit was used to create cDNA from one microgram of each total RNA sample (High-Capacity cDNA Reverse Transcription Kith ThermoFisher, USA). Quantitative PCR: For each of the 96 Real-Time ready Human Apoptosis Panels, a single cDNA synthesis experiment employing 5 g of total RNA as the template was carried out (Table 1). The total PCR reaction volume per well with Step-One Plus RT-PCR System (ThermoFisher, USA) was 20 μL. Sample setup and analysis was performed using the macro file for each gene panel with Step-One Plus Software v2.3.

### 2.9. Mammosphere (stem cell-enriched population) culture from T-47D cells

Cells from the human breast cancer cell line (parental) with CSCs characteristics and capable of forming mammosphere (sphere structures) were replicated in cell culture until the appropriate number was reached in order to propagate. The cell line was cultured in DMEM/F-12 medium containing 10% heat-inactivated fetal bovine serum (FBS) at 37 °C in 5% CO_2_. As the first step in obtaining stem cells, the spheroid structures, which we define as the mammosphere, were multiplied and collected. Floating cells known as “floating cells”, which are known to have stem cell characteristics, were collected from the appropriate number of human breast cancer cell lines. Collected cells were cultured in “ultralow attachment” cell culture dishes in DMEM-F12 growth medium containing 1X concentration of B27 Supplement w/o vitamin A, 0.2% heparin, 0.2% primocin.

### 2.10. Detection of stemness markers

Flow cytometry was used to assess the ratios of surface markers indicative of CD44^+^/CD24^−^ CSCs in order to identify CSCs (Erturk et al., 2021). For this purpose, a cell suspension was prepared from cells grown in a 75 cm^2^ “ultralow attachment” culture dish. After centrifugation, cells were labeled with staining antibodies (FITC antihuman CD44 Antibody, PE antihuman CD24 Antibody, BioLegend, San Diego, USA). After the cell solution was incubated with antibodies for 20 min, CD44^+^/CD24^−^ cell ratios were analyzed in Flow Cytometry (BD FACSCanto Flow Cytometer).

### 2.11. ATP viability assay in mammospheres

T-47D were seeded in 96-well cell culture plates at a density of 5 × 10^3^ cells per well in 100 μL of medium. To generate mamosphere structures, cells were cultured for 3–4 days. Doses in the range of 50–3.12 μM were applied for 48 h of SM treatment in the mammosphere. Luminescence signal was measured on a luminometer (Bio-Tek, Multi-Mode Microplate Reader, USA) and the result was expressed as relative light units (RLU). The viability of the treated mammosphere was calculated based on the absorbance results of the ATP test using the formula viability (%) = 100 × (Sample Abs)/(Control Abs) with reference to untreated control mammosphere.

### 2.12. Fluorescent imaging for determination of cell death mode in mammosphere

Mammosphere cells were seeded in 96-well ultralow surface plates at a density of 5 × 10^3^ cells per well in 100 μL culture medium. SM treatment was applied to the obtained mammosphere at a serial dilution dose of 50–3.12 μM for 24 and 48 h. At the end of the incubation period, mammospheres were stained with a working solution of 5 μg/mL Hoechst 33342 and 1 μg/mL PI dyes evaluated under a fluorescence microscope.

### 2.13. SDS–PAGE and western blotting

Mamospheres treated with SM compound, apoptosis ((BCL-2, 1:1000, #4223, Cell Signaling Technology), (BAX, 1:1000, #41162, Cell Signaling Technology)), stem cell marker ((NANOG, 1:1000, #8822, Cell Signaling Technology), (OCT4, 1:1000, #2750, Cell Signaling Technology), (SOX2, 1:1000, #2748, Cell Signaling Technology)), related signaling pathway components were examined at protein level. Cells treated to 50 μM SM treatment for 24 h and untreated control cells were first lysed to reveal the proteins inside them. Western blot analysis was performed according to the protocol in method 2.6.

### 2.14. RT-PCR assay in mammospheres

Forty-eight hours after SM treatment, BCSCs were collected for RNA isolation. Gene expression analysis was performed according to the protocol in method 2.7.

### 2.15. Statistical analysis

All statistical analyses were performed using the Graphpad v9.0 computer package program. Percent vitality values were calculated using one-way analysis of variance (ANOVA), and if the significance was different, the difference groups were determined with the Tukey Honest Significance Test (HSD). The results of all analyses performed with 3 replicates were given with the mean and standard deviation. Statistically significant data were determined according to the p < 0.05 value. IC_50_ values were calculated using Graphpad v9.0 software.

## 3. Results and discussion

### 3.1. Cytotoxic effects of SM on the viability of breast cancer cells

After 24-/48-h treatments, cytotoxic effects on cell viability of 1, 10, and 100 μM doses of SM compound were evaluated. It was observed that the amount of intracellular ATP decreased depending on the increase in the concentration of the SM compound and the treatment time. The ATP test result obtained when T-47D cells were treated with SM for 24 and 48 h is shown in Figure 1B. Based on the ATP results in T-47D cells, approximately 63% cell viability is seen at a concentration of 10 μM SM after 24 h of treatment. At the 100 μM concentration, a significant reduction in cell viability of up to approximately 5% is observed. After 48 h, it was observed that both 10 and 100 μM SM concentrations significantly affected cell viability and reduced cell viability below 15% (p < 0.001) (Figure 1B).

The IC_50_ value calculated for 24 h of treatment according to the ATP cell viability method is 31.15 μM, and the IC_50_ value calculated for 48 h is 6.18 μM concentration. IC_50_ values calculated according to the data obtained from SM application and ATP viability test are given in Table 2.

Considering other studies, Logashenko et al. calculated IC_50_ values of SM in KB-3-1 cell line are 0.3 μM for KB-3-1 cells, 1.2 μM for KB-8-5 cells, 1.3 μM for HeLa cells, and 0.8 μM for SK-N-MC (Logashenko et al., 2011). Also, Li et al. reported the IC_50_ dose as 0.36 μM for the HL-60 leukemia cells in their study (Li et al., 2014). In conclusion, the cytotoxic activity of the SM compound on cancer cells is promising. For MDCK and A549 cells, it is clear that SM at concentrations up to 2 μM and 1.5 μM, respectively, is not toxic to cells. The effects of SM toxicity were detected in these two cell lines only from higher concentrations (Markov et al., 2017).

### 3.2. Apoptosis inducing effect of SM

**Figure 2:**
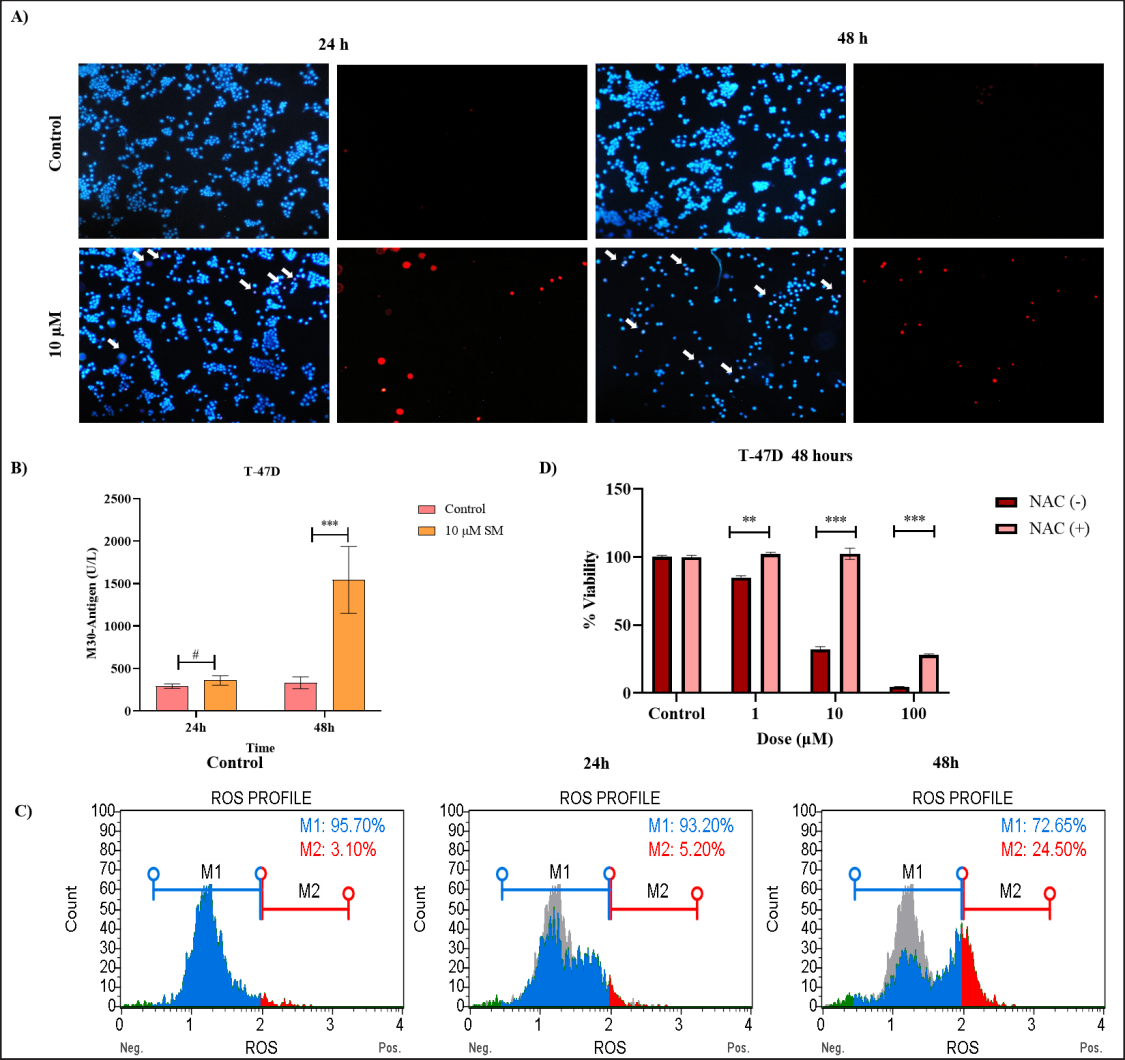
(A) Phase/contrast and fluorescence microscopes images of T-47D breast cancer cells stained with Hoechst 33342 (blue) and Propidium iodide (red) and treated with 10 μM SM for 24 and 48 h. Data were presented as mean ± SD (n = 2). (B) Detection of M30-antigen levels after 10 μM dose of treatment with SM for 24 and 48 h. #Denotes statistically significant differences between groups (Control and 10 μM SM): *** (p < 0.001), # no significant. Data are presented as mean ± SD (n = 3). (C) Flow cytometry of T-47D cells treated with 10 μM SM for 24 h and 48 h: Analysis results of oxidative stress assay. (D) Viability analysis results of T-47D cells inhibited by NAC and treated with SM for 48 h.

To investigate the cell death mode, the fluorescent staining method was applied as we show in Figure 2A. In Hoechst 33342 staining, the feature of having smaller nuclei than normal cells is sought in apoptotic cells treated with SM for 24 and 48 h. In necrotic cells, however, the nucleus should be slightly larger than in normal cells. Another apoptosis morphology seen in Hoechst 33342 staining is fragmented nuclei. Fragmented nuclei represent advanced stages of apoptotic cells. Images taken under fluorescence microscopy after 24 and 48 h of incubation following the application of 10 μM SM to T-47D cells is shown in Figure 2A. After 24 h of treatment, 63% of cells stained with Hoechst 33342 and PI stain were PI negative. This indicates that the cells are in the early stage of apoptosis. After 48 h of treatment, it is seen that the number of PI positive cells increased by 78% compared to 24 h of treatment. The increase in the number of PI positive cells indicates that the cells entered the advanced stages of apoptosis (early/secondary necrosis) after 48 hours of treatment. Fluorescence staining results showed statistically significant differences when SM-treated T-47D cells were compared with untreated control (p < 0.001). In another study, Hoechst 33342 staining was performed to examine the effect of 18βH-GA on SiHa human uterine carcinoma cells. Condensation and fragmentation, which are accepted as apoptotic markers, were detected in the nucleus of the SiHa cells after Hoechst 33342 staining (Lee et al., 2008).

Apoptosis was confirmed using the M30-antigen ELISA technique. The M30-antigen levels were calculated in U/L after the measured absorbances were reviewed over the formulas obtained with the use of the standard curve graph (Figure 2B). After 24 h of 10 μM dose of SM treatment in T-47D cell line, there was no increase in M30-antigen levels compared to control cells. There was a considerable rise in M30-antigen levels in T-47D cells after 48 h of treatment as compared to control cells (Figure 2B). In our previous study, Alper et al. (2021) discovered that MCF-7 cells treated with 24-h SM therapy had no rise in M30-antigen levels compared to the untreated control, but that after 48 h of treatment, M30-antigen levels had increased significantly (Alper et al., 2021).

**Figure 3:**
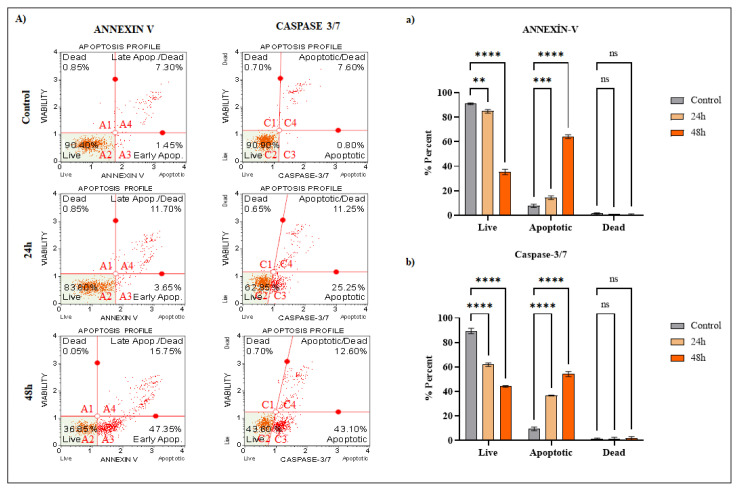
(A) Flow cytometry of T-47D cells treated with 10 μM SM for 24 and 48 h: Analysis results of annexin V and caspase 3/7. (a) Gradhpad statistical results of annexin V flow cytometry analysis. (b) Gradhpad statistical results of caspase 3/7 flow cytometry analysis. *Indicates statistically significant differences compared to untreated control: ****(p < 0.001). ns; indicates statistically insignificant differences compared to the untreated control. Data are presented as mean ± SD (n = 3).

Since SM was found to induce apoptosis, flow cytometry analysis was undertaken to establish apoptotic parameters. T-47D breast cancer cells were treated with 10 μM SM for 24 and 48 hours. The Muse Cell Analyzer was used to validate apoptotic cell death by measuring annexin V staining, caspase 3/7 activity, and ROS generation. Results from the Annexin V/7-AAD assay showed that T-47D cells increased the proportion of apoptotic cells to 15.35% (A3+A4) after 24 hours of treatment with the SM compound. After 48 h of SM treatment, apoptotic cells constitute 63.1% (A3+A4) of the total cell population (Figure 3). In addition, caspase 3/7 activation measurement results showed that apoptotic cells constituted 55.7% (C3+C4) of the total population in T-47D cells of 48 h of SM treatment (Figure 3A). Annexin V and caspase 3/7 flow cytometry results showed statistically significant differences when SM-treated T-47D cells were compared with untreated control (p < 0.001) (Figures 3a and 3b). When we look at other studies that have been studied with SM, Haghshenas et al. demonstrated that 18βH-GA triggered apoptosis on ovarian cancer cells as a result of annexin V-FITC measurement (Haghshenas et al., 2014). In another study, the effect of SM epoxides on caspase-3/−7 activity in B16 cells was investigated. As expected, at 1 μM, both ɑO-SM and βO-SM increased caspase-3/−7 activity approximately 2-fold in cells of similar potency compared to control. In addition, double staining with annexin V-FITC and PI was applied to B16 melanoma cells treated with new epoxides of SM. It showed that ɑO-SM and O-SM both produced an accumulation of around 74% with a similar efficiency when they caused apoptosis (Salomatina et al., 2022).

ROS is expressed in cells as a byproduct of the normal course of life. However, when excessive ROS production occurs, it causes significant damage such as DNA damage and mitochondrial disorders that leads to cell death (Pelicano et al., 2003; Ricci and Zong, 2006). Our results show that ROS production of T-47D cells increased by up to 24.5% (M2), especially after 48 h of SM treatment (Figure 2C). This indicates that T-47D cells are under oxidative stress. The determination of superoxide radicals is the basis of the kit used in flow cytometry research. As a result, superoxide radicals have been found to decrease in T-47D cells, although the total amount of ROS may increase. In Cai et al.’s study that supports our results, it was proven that 18βH-GA increased ROS levels in MDA-MB-231 breast cancer cells (Cai et al., 2017). In a study with soloxolone amides, soloxolone tryptamide showed high antiproliferative potential in glioblastoma cells. Soloxolone tryptamide appeared to induce ROS-dependent and autophagy-independent death of U87 and U118 glioblastoma cells via mitochondrial apoptosis (Markov et al., 2022). It has been demonstrated that 18p-glycyretinic acid causes HL60 cells to undergo apoptosis by increasing the generation of reactive oxygen species (Makino et al., 2006). ROS levels are decreased by 2-cyano-3,12-dioxooleana-1,9(11)-diene-28-oic acid (CDDO) and its methyl-ester, intracellular GSH, one of the most significant scavengers of ROS. The method by which CDDOs, derivatives of natural triterpenoids, exercise their cytotoxic effects is the fast and selective decrease of mitochondrial GSH (mGSH), which results in caspase-independent apoptosis (Borella et al., 2019).

The antioxidant N-acetylcysteine (NAC), a sulfhydryl group donor, contributes to the inhibition of oxidative stress (Tian et al., 2006). This effect is probably mediated by a NO-dependent mechanism (Pechánová et al., 2006). This protective mechanism is exerted by the inhibition of BH 4 oxidation by increased superoxide (Zembowicz et al., 1993). In addition, this molecule may protect against oxidative damage by inhibiting lipid peroxidation and scavenging ROS (De la Fuente and Victor, 2001). Our results show that T-47D cells increase ROS production by up to 24.5%, especially after 48 h of SM treatment. This indicates that T-47D cells are under oxidative stress. To demonstrate that cell death is caused by ROS, we applied an antioxidant, NAC, to our T-47D cells before SM treatment. We observed significantly higher viability in our NAC-treated T-47D cells 48 h after treatment compared to our non-NAC-treated T-47D cells (Figure 2D). After SM application, NAC scavenged ROS, protecting cells against oxidative damage. Markov et al. showed that U87 cells treated with NAC completely abolished the cytotoxic effect of the new soloxolon amides. This clearly shows that it is dependent on ROS (Markov et al., 2022).

### 3.3. Effect of SM on apoptotic proteins

**Figure 4:**
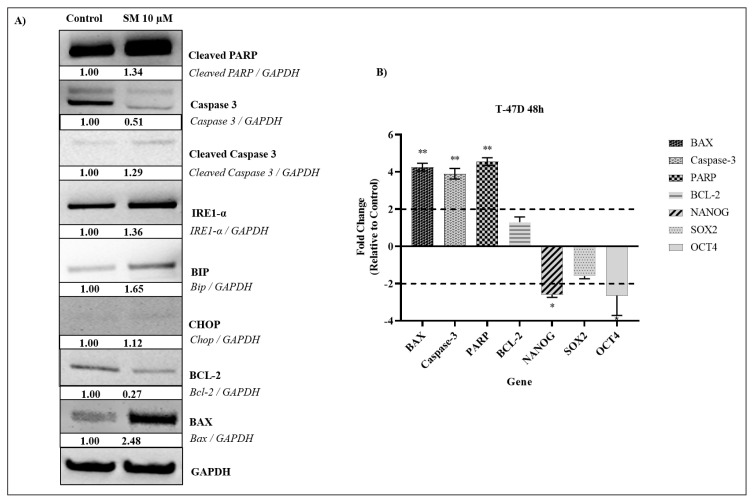
(A) Examination of apoptosis and ER-stress markers to elucidate apoptosis pathway. With the use of the ImageJ program, densitometry was carried out. GAPDH, or glyceraldehyde 3-phosphate dehydrogenase, was used as a reference point for quantification of the detected bands’ intensity. (B) Changes in the gene expression profiles of T-47D, determined by the simultaneous PCR method, after 48 h of treatment of SM compound with IC_70_ doses determined according to the ATP method. *Denotes statistically significant differences in comparison with control: *(p < 0.05), **(p < 0.01). Data are presented as mean ± SD (n=3). GAPDH, glyceraldehyde 3-phosphate dehydrogenase; RT-qPCR, quantitative real-time polymerase chain reaction; SD, standard deviation.

Western blotting was used to examine the changes in the apoptotic protein levels in the T-47D cell line caused by the SM compound treatment. Therefore, the IC_70_ (dose that kills 70% of the cells) calculated according to the ATP viability test was applied for 24 hours. According to the findings, when compared to untreated control cells, the expression level of cleaved PARP, cleaved Caspase-3 and BAX, which was used as an apoptotic marker in the T-47D cell line, was enhanced. The quantity of overall caspase-3 protein was reduced due to the rise in the amount of cleaved caspase-3 protein. BCL-2, an antiapoptotic protein, was shown to be present in lower amounts in direct proportion to the results acquired (Figure 4A). After the PARP protein is cleaved, it is cleaved into 24 kDA p24 N-terminal subunit and 89 kDA p89 C-terminal fragment. The capacity of the N-terminal region to bind strongly to DNA is not revealed until PARP is cleaved, but after PARP is cleaved by caspases, it binds irreversibly to the ends of DNA (Smulson et al., 1998). This inhibits poly ADP-ribose synthesis, thus eliminating the Base Excision Repair mechanism completely (D’Amours et al., 2001). There was also an increase in IRE1-α, BİP, CHOP proteins, which are associated with ER stress. The ATF-6 protein found in the ER has a stress-sensitive region in the C-terminal part of the ER lumen and an N-terminal region containing lysine in the cytoplasm-facing part that acts as a transcription factor (Numazawa et al., 2008). BİP is found bound to ATF-6 and IRE1-α under nonstress conditions. In stress situations, with the separation of BİP from ATF-6 and IRE1-α, the cytoplasmic region of ATF-6 enters the nucleus and initiates transcription. At the same time, IRE1-α becomes active by dimerization and autophosphorylation (Zhang and Kaufman, 2004; Scheuner and Kaufman, 2008). The IRE1-α arm is the best conserved in the evolution and the first signal arm of the unfolded protein response (UPR) signal network found in molecular terms (Schröder and Kaufman, 2005; Marciniak and Ron, 2006). The JNK signaling pathway is activated when the kinase domain of IRE1-α is activated (Malhotra and Kaufman, 2007; Urano et al., 2000). In summary, in cases where cells cannot cope with stress, receptors on the ER membrane such as IRE1-α can trigger apoptosis.

Considering these molecules, it is thought that IRE1-α and BİP go to apoptosis pathway via JNK signaling pathway via TRAF2. Because caspase-3 is a frequent effector in most apoptotic pathways and can cleave a variety of target proteins, this breakdown adds to the cell death program’s execution phase (de Moraes et al., 2011). It makes us think that the increase in ROS in cells stimulates the rapid translocation of Bad to mitochondria and accordingly there is a decrease in Bcl-2 expression. ROS increase also induced translocation of cytochrome c from mitochondria to cytosol. This is indicative of mitochondrial dysfunction. These changes resulted in an irreversible loss of mitochondrial membrane potential. These data suggest that the regulation of Bcl-2 and mitochondrial function are important factors in oxidative stress-induced apoptosis (Cook et al., 1999). As a result, there is an increase in active caspase-3 levels by ER-stress that can cause activation of caspase-3. Caspase-3 activation can directly lead to cleavage of PARP so it binds irreversibly to the ends of DNA and can cause the cell to undergo apoptosis.

When we look at the studies, BİP, PERK, ERP72, which are proteins associated with ER stress of Glycyrrhetinic acid (GA) on A549 and H460 lung cell lines, were examined and it was determined that GA causes ER stress due to the increases in these proteins (Zhu et al., 2015). In another study, significant increases in CHOP and IRE1-α proteins were observed in MDA-MB-231 cells treated with capsaicin, resulting in ER stress-mediated apoptosis with the expression of caspase-7 (Choi et al., 2010).

### 3.6. Gene expression changes caused by SM in T-47D

Target genes were identified and their expression levels were evaluated by RT-qPCR. Expression analysis showed that 7 different genes (BAX, Caspase-3, PARP, BCL-2, NANOG, SOX2, and OCT4) were expressed at different levels compared to control cells after SM treatment. Overall, these genes were chosen because they have important roles in cell death and are relevant to CSCs biology. An expression study of 7 genes compared with control cells after SM treatment showed that some genes were expressed at different levels. Three genes (BAX, Caspase-3 and PARP) were up-regulated in T-47Ds from 1.950 to 227-fold over 48 h. In addition, 3 genes (NANOG, SOX2 and OCT4) were downregulated between 1.30 and 1.32-fold. RT-qPCR analysis showed that expression levels of BAX, Caspase-3, and PARP genes were statistically increased in T-47D cells after 48 h of treatment (**p < 0.01). In addition, statistically significant decrease was observed in NANOG, SOX2, OCT4 gene expression levels in T-47D cells at 48th hour (*p < 0.05) (Figure 4B). It has been demonstrated that SM effectively suppresses the stem cell characteristics of T-47D cells by lowering the expression levels of key genes (NANOG, SOX2, and OCT4) that are involved in CSCs regulation. Additionally, it has been demonstrated that SM causes the overexpression of the cell death-related genes BAX, PARP, and Caspase-3 after 48 h of treatment. Increased BAX, Caspase-3, and PARP and decreased BCL-2 gene levels according to RT-PCR results indicate that SM treatment induces apoptosis cell death in T-47D cell line.

Markov et al. (2019) looked at differentially expressed genes (DEGs), which comprised 362 genes with an expression change of 1.5-fold or more during the first 1–4 h after SM therapy in order to better understand the SM mechanism of action. Seventeen genes were found to be shared across the entire early phase of SM action among the discovered DEGs. It regulates cellular metabolism (ADSSL1, MSMO1, PFKFB4, AGXT2L1), nuclear organization (LBR, HIST1H2BD), gene expression (APP, AHCY), Ca2+ signaling (CXCR4), protein glycosylation (DPM1), intracellular transport (GOLIM4, RAB17) and it contained downregulated DEGs that control γ-aminobutyric acid transport (SLC6A12). Widespread upregulation of DEGs was linked to the control of actin cytoskeleton organization and stress-induced protein folding (HSPA6, CAPZB). It was discovered that important intracellular processes start to occur after 4 h of SM therapy and that the cellular stress caused by SM in its early phases is typically related to its impact on the ER (Markov et al., 2019).

### 3.4. Cytotoxic effects of soloxolone methly on BCSCs viability

**Figure 5:**
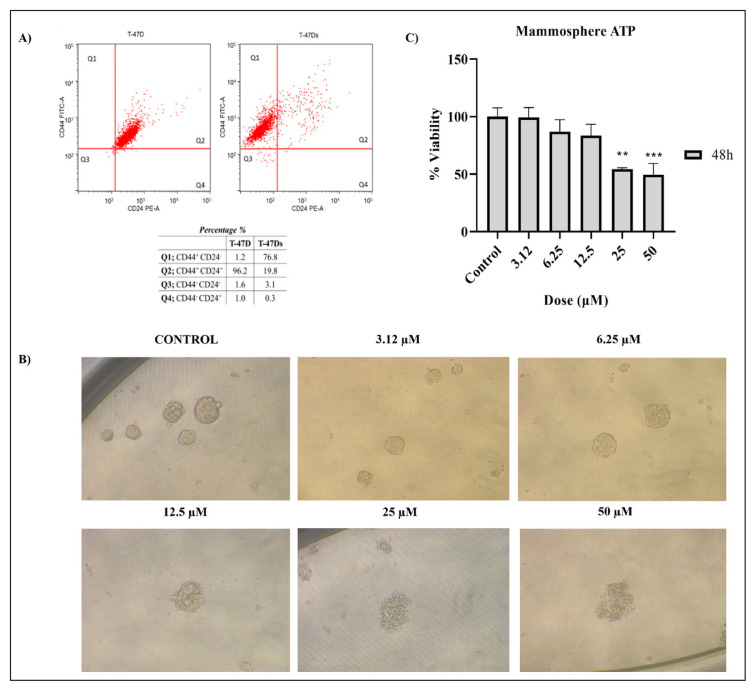
(A) Flow Cytometry results of CD44 and CD24 cells ratios of T-47D human breast cancer stem cell-like cells. (B) Phase-contrast microscope photographs of mammosphere cells after 48 h of 3.12–50 μM doses of SM treatment. (C) Viability of mammosphere cells treated with 3.12–50 μM doses of SM for 48 h. ATP assay were performed as cell viability assays. *Indicates statistically significant differences compared to untreated control: **(p < 0.01), ***(p < 0.001). Data are presented as mean ± SD (n = 3).

In our study, CD44+/CD24− cancer stem-like cells in T-47D cells with surface markers were evaluated by flow cytometry. It was determined that CD44+/CD24− cells ratio measured 76.8% in mammosphere (Figure 5A).

After 48-h application of 3.12–50 μM doses of SM compound to mammosphere cells, cytotoxic effects on cell viability were evaluated with phase-contrast microscope images and ATP assay results. Phase-contrast microscope images and ATP test results obtained when mammosphere cells were treated with SM for 48 h are shown in Figures 5B and 5C. When the phase-contrast microscope images are examined, it is seen that the spheroid structure is disrupted at 12.5 μM and higher doses (Figure 5B). When the ATP test results are examined, it is observed that different concentrations 3.12–50 μM doses of the SM compound cause a decrease in the amount of intracellular ATP levels. According to the ATP results in mammosphere cells, a significant decrease in cell viability was observed at both 50 and 25 μM doses after 48 h of treatment (p < 0.01**, p < 0.001***) (Figure 5C). The IC_50_ value calculated for 48 h based on the ATP method is 47.77 μM dose. IC_50_ values calculated according to the data obtained from SM application and ATP viability test are given in Table 2. In conclusion, the cytotoxic activity of the SM compound on mammosphere cells is at least as promising as the effect observed in T-47D cells. According to the literature review, no CSCs studies have been carried out with SM, 18βH-GA, or 18βH-GA derivatives.

### 3.5. Apoptosis-inducing effect of SM on BCSCs

**Figure 6:**
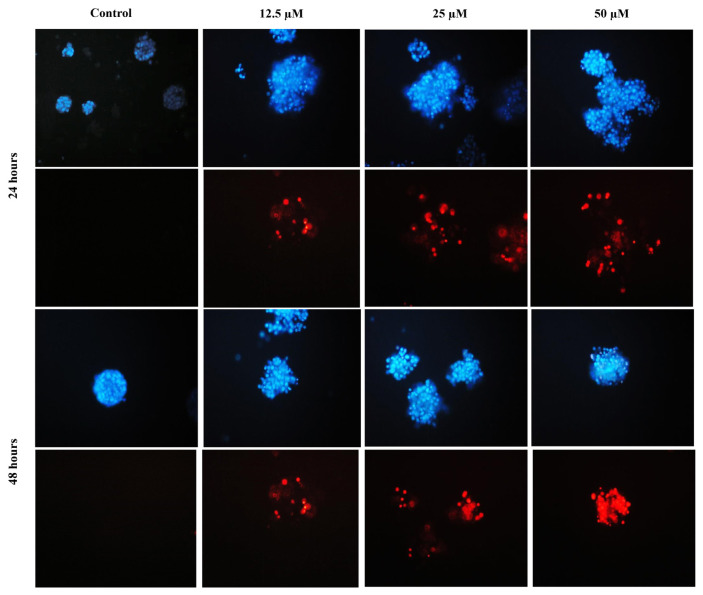
Fluorescence microscopes images of BCSCs stained with Hoechst 33342 (blue) and Propidium iodide (red) and treated with 50–12.5 μM doses of SM for 24 h. Data were presented as mean ± SD (n = 2).

Fluorescent staining method was applied after 24 and 48 h treatments in mammosphere cells to investigate the cell death mode. Following the application of 3.12–50 μM doses of SM to mammosphere cells, images taken under fluorescence microscope after 24 and 48 h of incubation are shown in Figure 6. As a result of 24 h application with SM, cell nuclei were pycnotic and fragmented compared to control cells, and PI positivity was not observed in approximately 85% of the cells. Therefore, it can be said that these cells are in the early stage of apoptosis (p < 0.01) (Figure 6). However, according to the 48 h treatment results, 45% of the regions in which pycnotic nuclei were observed with Hoechst staining in mammosphere cells were PI-positive (p < 0.001) (Figure 6). Accordingly, the visible increase in pycnotic/fragmented nuclei and PI-positive cells after 48 h of treatment compared to 24 h shows us that mammosphere cells have late apoptosis/secondary necrosis. In addition, it is observed that the number of pycnotic/fragmented nuclei and PI-positive mammosphere cells increases as the dose increases in both 24 h and 48 h SM treatment.

### 3.6. Effect of SM on apoptotic proteins in the mammosphere

**Figure 7:**
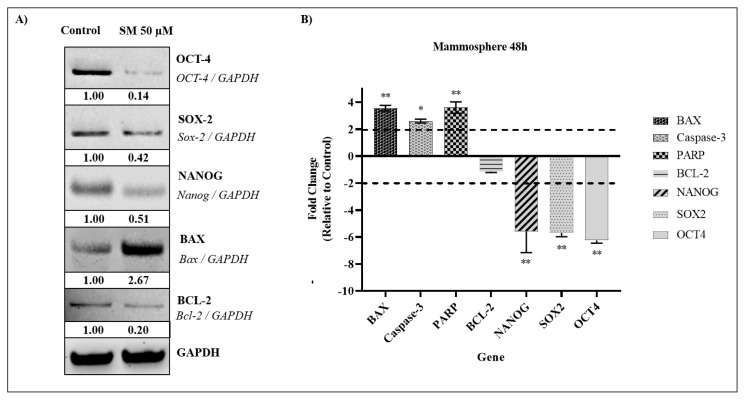
(A) Examination of apoptosis and ER-stress markers to elucidate apoptosis pathway. With the use of the ImageJ program, densitometry was carried out. GAPDH, or glyceraldehyde 3-phosphate dehydrogenase, was used as a reference point for quantification of the detected bands’ intensity. (B) Changes in the gene expression profiles of BCSCs, determined by the simultaneous PCR method, after 48 h of treatment of SM compound with IC_70_ doses determined according to the ATP method. *Denotes statistically significant differences in comparison with control: *(p < 0.05), **(p < 0.01). Data are presented as mean ± SD (n = 3). GAPDH, glyceraldehyde 3-phosphate dehydrogenase; RT-qPCR, quantitative real-time polymerase chain reaction; SD, standard deviation.

Western analysis was used to assess the amounts of stem cell and apoptotic proteins of CSCs derived from breast cancer cells. To examine changes in apoptotic protein levels in the mammosphere caused by SM compound treatment, IC_70_ (dose that kills 70% of cells), calculated according to the ATP viability test, was administered for 24 h. According to the findings, the expression level of BAX, which is used as an apoptotic marker in the mammosphere, increased compared to the untreated control cells. The decrease in the level of BCL-2, an antiapoptotic protein, proved to us once again apoptosis. In addition, significant decreases were observed in NANOG, SOX2, OCT4 protein levels in the mammosphere. The decrease in the expression of these proteins means that SM suppresses stem cell properties in the mammosphere (Figure 7A).

### 3.7. Gene expression changes caused by SM in BCSCs

The expression levels of genes targeted in the gene expression analysis of T-47D cells in CSCs derived from breast cancer cells were also evaluated by RT-qPCR. Expression analysis showed that 7 different genes (BAX, Caspase-3, PARP, BCL-2, NANOG, SOX2, and OCT4) were expressed at different levels in the mammospheres compared to control cells after SM treatment. Three genes (BAX, Caspase-3 and PARP) were upregulated in the mammospheres from 1.30 to 2.15-fold at 48 h. In addition, 3 genes (NANOG, SOX2, and OCT4) were downregulated between 2.80 and 3.12-fold. RT-qPCR analysis showed that expression levels of BAX, Caspase-3, and PARP genes were statistically increased in the mammospheres after 48 h of treatment (*p < 0.05, **p < 0.01). In addition, statistically significant decrease was observed in NANOG, SOX2, OCT4 gene expression levels in the mammosphere at the 48th hour (**p < 0.01) (Figure 7B). It has been observed that SM also suppresses stem cell properties in the mammosphere and the treatment is effective. The gene expression results analyzed after SM treatment of mammospheres and T-47D cells are in support of each other.

## 4. Conclusion

SM substance, a semisynthetic GA derivative derived from the *Glycyrrhiza glabra* (licorice root) plant, was demonstrated in this work to have substantial cytotoxic activity in T-47D human breast cancer cell lines and BCSCs. It was found that SM exhibited cytotoxic activity and induced apoptosis in both T-47D cell line and mammmospheres. The therapy was successful in suppressing the stem cell characteristics of T-47D cells and mammospheres as evidenced by the lower expression levels of genes that are important in the control of CSCs. The fact that the SM compound has not been studied on CSCs makes our study valuable. Despite the promising findings of this in vitro study, it was concluded that the next step should be examined for the effect of in vivo conditions.

## Figures and Tables

**Table 1:** RT-PCR primer list.

Gene	Forward	Reverse
**BAX**	5′-CTACAGGGTTTCATCCAG-3′	5′-CCAGTTCATCTCCAATTCG-3′
**Caspase-3**	5′-GTGGAACTGACGATGATATGGC-3′	5′-CGCAAAGTGACTGGATGAACC-3′
**PARP**	5′-TGACCAGCAGAAAGTCAAGAA-3′	5′-CAAAGTCACCCAGAGTCTTCTC-3′
**BCL-2**	5′-GTGGATGACTGAGTACCTGAAC-3′	5′-GAGACAGCCAGGAGAAATCAA-3′
**NANOG**	5′-TCCTGAACCTCAGCTACAAAC-3′	5′-GCGTCACACCATTGCTATTC-3′
**SOX2**	5′-CACCTACAGCATGTCCTACTC-3′	5′-TGGGAGGAAGAGGTAACCA-3′
**OCT4**	5′-GGAGGAAGCTGACAACAATGA-3′	5′-CTCTCACTCGGTTCTCGATCAT-3′
**GAPDH**	5′-GCTCTCTGCTCCTCCTGTTC-3′	5′-ACGACCAAATCCGTTGACTC-3′

**Table 2:** IC_50_ values of T-47D cells and mammosphere treated with SM according to ATP assay results.

Complex	Time (h)	Test type	IC_50_ (μM)
T-47D	24	ATP	31.15
T-47D	48	ATP	6.18
Mammosphere	48	ATP	47.77

* **IC_50_** is defined as the dose inhibiting 50% of viability.

## Data Availability

No data was used for the research described in the article.
